# Screening of TNF signaling pathway-related genes in knee osteoarthritis (KOA) using WGCNA and machine learning

**DOI:** 10.1097/MD.0000000000043849

**Published:** 2025-08-15

**Authors:** Shaoyang Zhai, Rui Wu, Shengzhen Fan, Ge Du, Xinkun Zhao, Haoran Wan, Weichen Huang

**Affiliations:** a Orthopedic Injury College, Guizhou University of Traditional Chinese Medicine, Guiyang, Guizhou, China; b The Second School of Clinical Medicine Guizhou University of Traditional Chinese Medicine, Guiyang, Guizhou, China; c Joint Orthopedics, The Second Affiliated Hospital of Guizhou University of Traditional Chinese Medicine, Guiyang, Guizhou, China.

**Keywords:** bioinformatics, knee osteoarthritis, machine learning, TNF signaling pathway, WGCNA

## Abstract

Knee osteoarthritis (KOA), a prevalent degenerative joint disease, involves complex inflammatory responses and signal pathway regulation in its pathogenesis. This study investigates the tumor necrosis factor signaling pathway’s mechanistic role in KOA using diverse bioinformatics approaches. Initially, differential expression analysis identified 222 significant differentially expressed genes. Subsequent Gene Ontology and Kyoto Encyclopedia of Genes and Genomes enrichment analyses indicated that these differentially expressed genes are mainly involved in inflammatory responses and cytokine signaling pathways. Weighted gene co-expression network analysis was then employed to identify the MEbrown module, which is closely associated with knee arthritis, and 20 key tumor necrosis factor signaling pathway-related genes were selected. Further analysis using LASSO regression and the Boruta algorithm screened out important genes, including MCL1, JUN, and GADD45B, whose superior performance in classification tasks was validated. Additionally, immune infiltration analysis revealed significant differences in immune cell distribution across knee arthritis samples, underscoring the critical role of inflammation regulation in disease progression. Overall, this study enhances the understanding of KOA pathogenesis and offers valuable insights for personalized treatment and, precision medicine.

## 1. Introduction

Knee osteoarthritis (KOA), a degenerative condition, primarily manifests through the deterioration of knee cartilage, the growth of bone spurs, and inflammation within the joint.^[1]^ It stands as one of the predominant types of osteoarthritis (OA), comprising over 80% of all OA cases.^[2,3]^ Global disease burden studies indicate that knee osteoarthritis is a major contributor to disability worldwide, particularly affecting the aging population whose incidence rates escalate with advancing age.^[1]^

There are 2 principal classifications of knee osteoarthritis: primary and secondary. The former is usually linked to aging and genetic predispositions, whereas the latter often results from injuries, obesity, or metabolic disorders.^[4–6]^ The progression of KOA is influenced by various factors. In patients with tibial plateau fractures, factors such as age and BMI can significantly impact functional outcomes. Postoperative articular step-offs and tibial axis malalignment may cause pain. Furthermore, certain fracture types are prone to post-traumatic osteoarthritis. These factors highlight the post-traumatic nature of KOA and demonstrate that its development results from the interplay of biomechanical and inflammatory processes. Synovitis is also common in KOA, involving multiple joint tissues, with the synovium playing a primary role.^[7,8]^ The diagnosis of this condition typically depends on radiological exams and evaluations of symptoms. As for treatment, it spans pharmaceuticals, physiotherapy, and surgical interventions, with recent focus shifting towards biological treatments such as inhibitors of tumor necrosis factor (TNF).^[9]^

The TNF signaling pathway is mediated by tumor necrosis factor-alpha (TNF-α) and its receptors, forming an intracellular signaling network. TNF-α, a key pro-inflammatory cytokine predominantly secreted by macrophages and fibroblasts, activates downstream signaling pathways such as NF-κB and mitogen-activated protein kinase through TNFR1 and TNFR2, playing a crucial role in inflammatory responses, cell apoptosis, and immune regulation.^[9–11]^ TNF-α drives cartilage degeneration by stimulating IL-1β and MMPs, which degrade aggrecan and type II collagen. This reduces cartilage elasticity and integrity. TNF-α also inhibits proteoglycan synthesis and induces chondrocyte apoptosis, accelerating cartilage thinning. These actions collectively worsen knee osteoarthritis progression.^[12,13]^ The TNF signaling pathway contributes to the pathogenesis of knee osteoarthritis through various mechanisms, including the promotion of inflammatory cytokine release, acceleration of cartilage matrix degradation, and stimulation of osteophyte formation. Studies have shown that elevated levels of TNF-α are closely associated with the degree of inflammation and disease severity in patients with knee osteoarthritis.^[14,15]^ The TNF pathway facilitates the degradation of the cartilage matrix by inducing the release of inflammatory mediators such as IL-1β and MMPs.^[16,17]^ Additionally, it regulates chondrocyte apoptosis and bone metabolism imbalance, exacerbating the progression of knee osteoarthritis.^[18]^ Research on genes like TNF, TNFR, and NF-κB has revealed their significant roles in knee osteoarthritis. For instance, the co-expression of TNF-α and MMP13 is considered a key driver of cartilage degradation,^[19]^ while NF-κB signaling activity is associated with chronic inflammation in knee osteoarthritis.^[9]^ TNF-α inhibitors such as etanercept and adalimumab have shown significant efficacy in treating rheumatoid arthritis, and their potential application in knee osteoarthritis is also receiving considerable attention.^[20,21]^ However, further research is needed to optimize dosages, clarify side effects, and explore new treatment combinations.

The rapid advancement of high-throughput sequencing technologies has provided strong support for the discovery of genes associated with knee osteoarthritis.^[22]^ Differential expression analysis, weighted gene co-expression network analysis (WGCNA), and machine learning are commonly used bioinformatics tools that effectively identify key genes and biomarkers for knee osteoarthritis.^[23,24]^ Additionally, these methods have achieved notable success in the study of diseases such as idiopathic pulmonary fibrosis, offering valuable insights for research into knee osteoarthritis.

Focusing on genes associated with the TNF signaling pathway, this study aims to explore their mechanisms roles in knee osteoarthritis. Through WGCNA and machine learning methods, we will screen for key genes in knee osteoarthritis and identify potential diagnostic and therapeutic targets. This study will deepen our understanding of the pathogenesis of knee osteoarthritis and provide a basis for personalized treatment and precision medicine.

## 2. Methods

### 2.1. Differential expression analysis of mRNAs

The mRNA expression profiles were obtained from the Gene Expression Omnibus database (https://www.ncbi.nlm.nih.gov/geo/) under accession number GSE55457 (Platform GPL96). This dataset includes 33 synovial tissue samples, of which 10 were normal samples and 13 were rheumatoid arthritis samples, with the remaining 10 being OA samples. Given the focus of this study on OA, only the normal and OA samples were selected for further analysis. TNF signaling pathway-related genes were retrieved from the GSEA database, totaling 200 genes (https://www.gsea-msigdb.org/gsea/msigdb/human/geneset/HALLMARK_TNFA_SIGNALING_VIA_NFKB).

The microarray data utilized in this study had been pre-processed by the data uploader, ensuring no anomalous probe data or missing values. However, experimental errors can still be introduced due to external environmental conditions during microarray sequencing, which may affect subsequent sample comparisons and calculations. Therefore, data normalization was performed to eliminate magnitude differences before any computations.

The “limma” R package was employed for differential expression analysis of the normalized dataset. Differentially expressed genes (DEGs) were identified based on the criteria of |log 2 fold change (FC)| > 1.5 and *P*-value < .05. Specifically, genes with log FC > 1.5 and *P*-value < .05 were considered upregulated, while those with log FC < ‐1.5 and *P*-value < .05 were regarded as downregulated. Volcano plots of the DEGs were constructed using the “ggplot2” package, and heatmaps were generated with the “ComplexHeatmap” package.

### 2.2. Functional enrichment analysis and PPI network construction

The Kyoto Encyclopedia of Genes and Genomes (KEGG) is a database for systematic analysis of gene functions, linking gene information to biological pathways. In this study, KEGG analysis was conducted to categorize genes into their corresponding functional pathways, which aids in exploring the relationship between differentially expressed genes in knee osteoarthritis and the TNF signaling pathway. Additionally, the Gene Ontology (GO) project provides ontologies to describe the attributes of gene products across 3 non-overlapping domains: molecular function, biological process, and cellular component. GO and KEGG enrichment analyses were performed using the “clusterProfiler” package in R software. Candidate genes were selected from the highly credible “TNF signaling pathway.”

Protein–protein interaction (PPI) analysis is a method used to study protein interactions. It reveals the molecular network structure within cells by connecting genes based on their corresponding protein interactions. In this study, the STRING search tool was employed to query the gene sets obtained from GO analysis. STRING is an online tool and database designed to display networks of PPIs, offering comprehensive insights into how proteins interact within the cell.

### 2.3. Weighted gene co-expression network analysis

In this study, the weighted gene co-expression network analysis was performed using the “WGCNA”package. At the initial stage of the analysis, outlier samples were strictly identified and removed to ensure the reliability and accuracy of the subsequent analysis data. For the remaining samples, the correlations among genes were accurately calculated, and a scale-free network was constructed based on these correlations. During the network construction, the soft threshold was continuously adjusted and verified until the scale-free fitting index (R²) approached 0.85. At this point, the soft threshold was determined to be the optimal choice, thus ensuring the adequate presentation of the network’s scale-free nature. Based on this, hierarchical clustering and a dynamic tree-cutting algorithm were used to scientifically divide all genes into different modules. Furthermore, using the occurrence of OA as a trait variable, an in-depth correlation analysis was carried out between each module and the OA disease status. After rigorous screening and evaluation, the modules most significantly associated with OA development were accurately identified. This provided a solid and reliable starting point for further investigating the pathogenesis of OA and identifying key genes involved in the disease.

### 2.4. Candidate genes obtaining

To identify key genes with significant biological relevance in knee osteoarthritis, we performed an intersection analysis of gene sets from 3 sources: DEGs, genes in the MEbrown module from the WGCNA, and TNF signaling pathway-related genes. The intersection of these 3 gene sets yielded a group of crucial genes that may play a significant role in the development and progression of knee osteoarthritis.

To further refine the selection of genes associated with knee osteoarthritis, a least absolute shrinkage and selection operator (LASSO) regression model was constructed. LASSO regression shrinks the coefficients of variables in the model, reducing some of the coefficients to zero, thereby performing variable selection and simplifying the model. Gene expression data was normalized to eliminate differences in gene expression levels. The “glmnet” package was employed to build the LASSO regression model. During training, cross-validation was used to select the optimal regularization parameter λ. Based on the best λ value, genes with non-zero regression coefficients were identified as candidate genes significantly associated with knee osteoarthritis.

The Boruta algorithm, a feature selection method based on random forests, was employed to select the most important features from the original feature set for predicting the target variable. It works by introducing random shadow features and comparing their importance with that of the actual features. If a feature’s importance is significantly higher than that of the random shadow features, it is considered to be significantly useful. Feature selection was carried out using the Boruta package in the R environment, and genes marked as green in the results were identified as significantly important features in this study. Finally, to integrate the results of the LASSO regression and Boruta feature selection, a final intersection analysis was performed. The candidate genes obtained from both methods were used to train the machine learning models. After training, the genes identified as important by both models were retained as the final set of key genes. This integration approach ensures that the selected genes are robust and have high predictive power for knee osteoarthritis.

### 2.5. Core gene validation and immune infiltration analysis

To evaluate the predictive ability of the selected genes in classification tasks, receiver operating characteristic curve analysis was performed using the R programming language and related packages. The area under the curve (AUC) value for each gene was calculated and presented graphically. The AUC values reflect the accuracy of the genes in validating knee osteoarthritis. Besides, immune cell infiltration in osteoarthritis tissues was analyzed using gene expression data from knee osteoarthritis and normal tissue samples. The CIBERSORT algorithm, a deconvolution method based on a gene expression signature matrix (LM22 matrix) for specific immune cell types, was used to estimate the immune cell composition. It quantifies the relative abundance of 22 immune cell subtypes. Normalized gene expression data were used as input, with low-expression genes filtered out to enhance analysis accuracy. The analysis was conducted using the online CIBERSORT tool or local R scripts.

## 3. Results

### 3.1. 222 DEGs were identified between OA and normal samples

**Figure 1.**
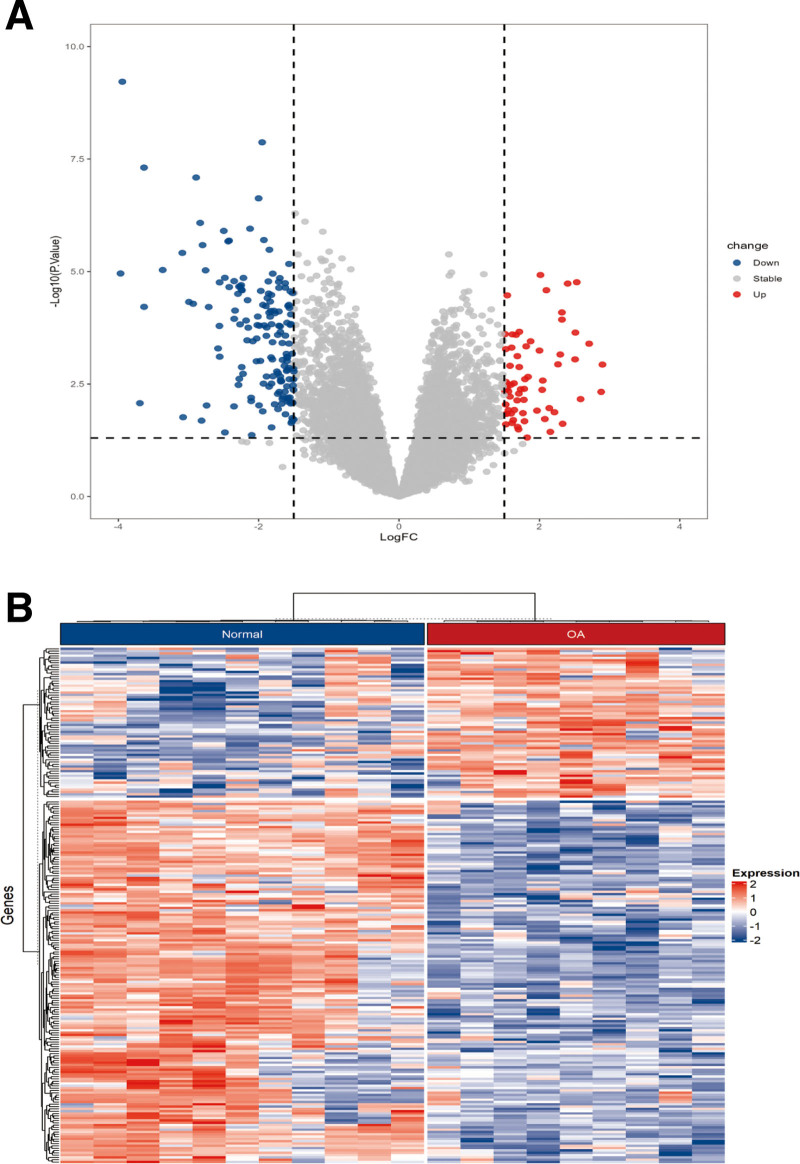
Differential expression analysis. (A) Volcano plot of DEGs in GSE55457. In the GSE55457 dataset, blue points represent downregulated genes, gray points indicate genes with no significant difference, and red points represent genes that are upregulated in the OA group compared to the normal group. (B) Hierarchical clustering heatmap of DEGs in GSE55457. DEG = differentially expressed genes, OA = osteoarthritis.

Differential expression analysis between OA samples and normal samples identified 222 DEGs (65 upregulated and 157 downregulated, *P* < .05, |log FC| > 1.5). These genes highlight significant expression differences between OA and normal samples. Volcano plots and heatmaps were used to visualize the DEGs. The volcano plot (Fig. 1A) shows the distribution of log FC and adjusted *P*-values for all genes, with red and green points representing significantly upregulated and downregulated genes, respectively. The heatmap (Fig. 1B) illustrates the expression patterns of significant DEGs across all samples, emphasizing the expression differences between OA and normal samples.

**Figure 2.**
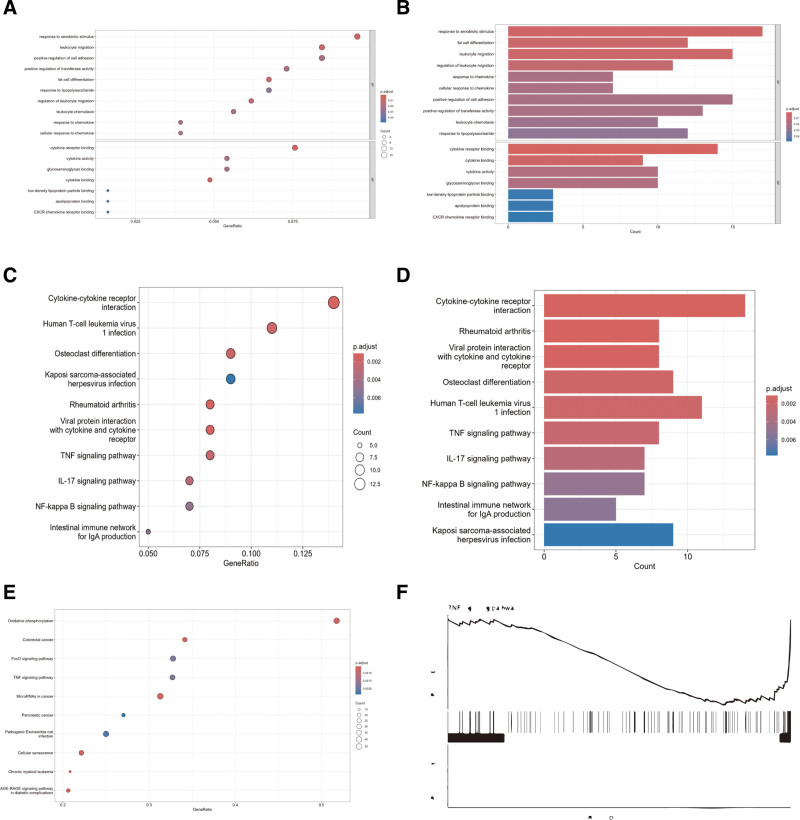
Functional enrichment analysis of differentially expressed genes. (A) Volcano plot of DEGs in GSE55457. In the GSE55457 dataset, blue points represent downregulated genes, gray points indicate genes with no significant difference, and red points represent genes that are upregulated in the OA group compared to the normal group. (B) Hierarchical clustering heatmap of DEGs in GSE55457. (C) Hierarchical clustering heatmap of DEGs in GSE55457. (D) Hierarchical clustering heatmap of DEGs in GSE55457. (E) Hierarchical clustering heatmap of DEGs in GSE55457. (F) Hierarchical clustering heatmap of DEGs in GSE55457. The descriptions for C, D, E, and F appear identical. If each heatmap corresponds to different sets of analyses or data categories, please specify those details for clarification. DEG = differentially expressed genes, OA = osteoarthritis.

GO and KEGG enrichment analyses were performed to explore the potential biological functions of the DEGs. The results are displayed in bubble plots and bar charts (Fig. 2A–F). GO enrichment revealed significant enrichment of DEGs in categories like response to xenobiotic stimulus (biological process) and cytokine receptor binding (molecular function), indicating close links to stress responses and cytokine receptor interactions. KEGG analysis showed that DEGs are mainly enriched in the cytokine–cytokine receptor interaction pathway, with significant enrichment also observed in the TNF signaling pathway. Analysis further supported the importance of the TNF signaling pathway, indicating that TNF-related genes are predominantly enriched in low-expression regions, suggesting potential suppression of this pathway under study conditions. This suppression might be closely associated with immune function regulation and inflammatory responses, offering key insights into disease mechanisms.

**Figure 3.**
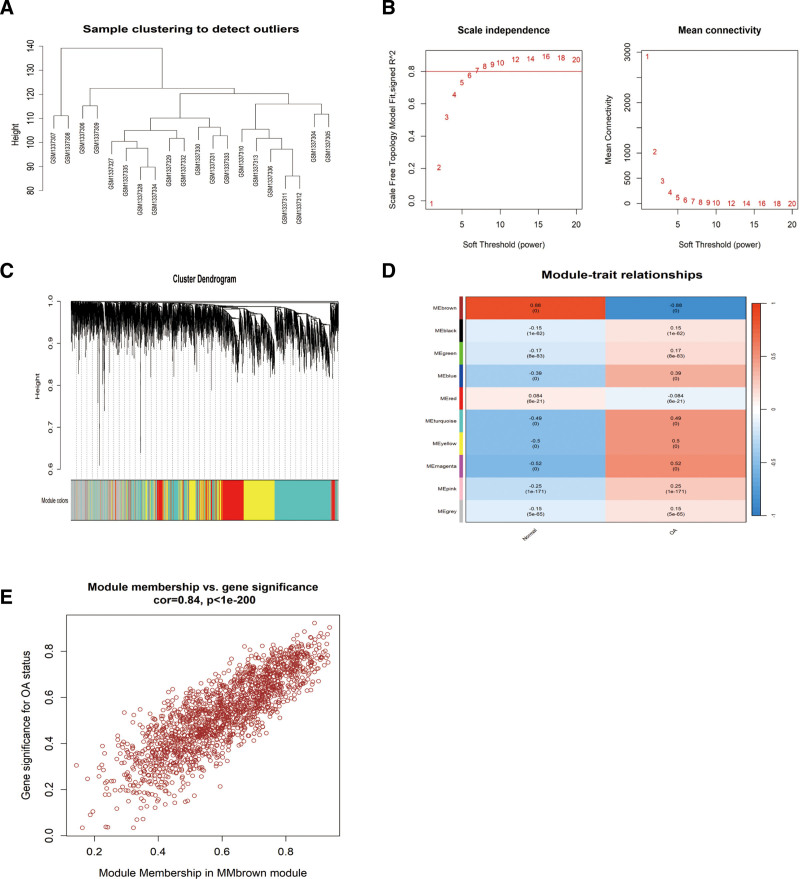
Weighted gene co-expression network analysis. (A) Dendrogram construction. Modules with similar expression profiles were merged. (B) Construction of the scale-free network in the co-expression network. Scale independence and average connectivity analysis (β = 10). (C) Dendrogram of differentially expressed genes based on dissimilarity measure (1-TOM) clustering. (D) Gene correlation scatter plot for the brown module. (E) Module membership was highly correlated with gene significance.

PPI network analysis of the DEGs resulted in a network with 200 nodes and 387 edges. Key genes in the network are significantly involved in biological processes such as response to endogenous stimulus and organic substance. Many of these key genes are closely related to OA development and progression. A PPI network diagram (Fig. 3) was constructed to highlight significant submodules, revealing strong interactions among key genes. These findings provide valuable insights for further functional and mechanistic studies.

### 3.2. WGCNA analysis results

To identify gene co-expression modules closely related to OA and Normal samples, we applied the WGCNA method. By analyzing the gene expression data from the samples, we identified several gene co-expression modules and performed a correlation analysis between these modules and the sample phenotypes. First, we used the goodSamplesGenes function to check for missing values in the data. The results indicated that no sample filtration was necessary (Fig. 3A). Next, we employed the pickSoftThreshold function to select and validate the optimal soft threshold, determining the power index β = 10 (Fig. 3B). Then, using the Pearson correlation coefficient, we constructed the adjacency matrix and converted it into the topological overlap matrix (TOM). The phase anisotropy (1-TOM) was calculated under the appropriate power index. Based on the TOM, genes were divided into 10 distinct modules (Fig. 3C). Finally, we selected the module most strongly correlated with the OA phenotype (normal and OA) for further analysis. The module MEbrown, which showed the strongest correlation with the OA phenotype (correlation coefficient = ‐0.88, *P* = 0), was chosen for further study (Fig. 3D). We then calculated the module membership, which showed a strong correlation (cor = 0.84, *P* < 1*e*‐200) (Fig. 3E).

### 3.3. Three core gene were screening through machine learning algorithms

**Figure 4.**
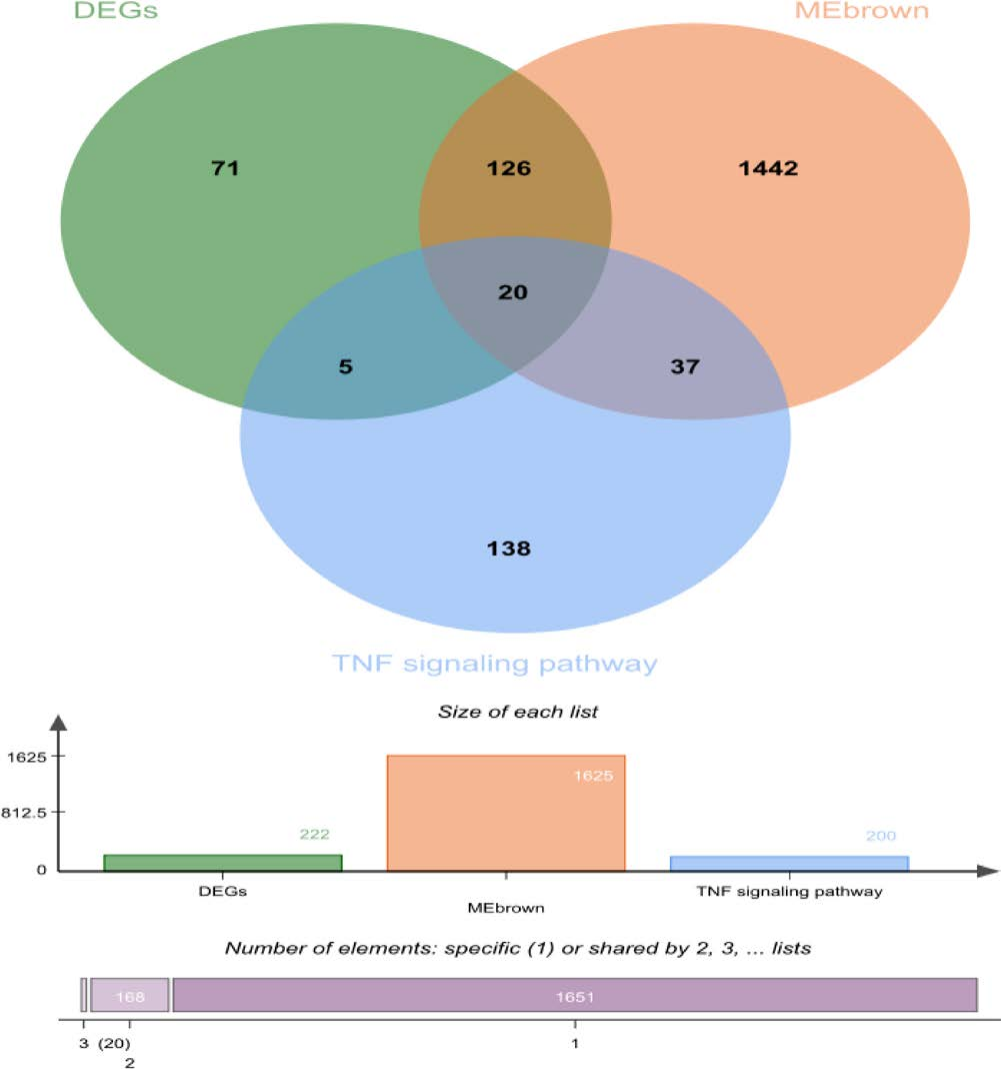
Venn diagram showing the intersection of DEGs, genes in the brown module, and TNF signaling pathway-related genes. DEG = differentially expressed genes, TNF = tumor necrosis factor, TOM = topological overlap matrix.

**Figure 5.**
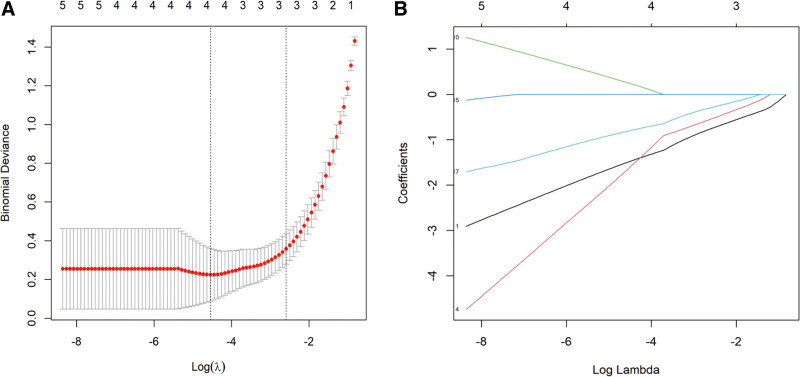
Gene selection using LASSO algorithm. (A) LASSO regression regularization path for DEGs. This plot illustrates the effect of different regularization strengths (λ) on the model’s misclassification rate. The *x*-axis represents the logarithm of the regularization parameter (log(λ)), and the *y*-axis represents the misclassification rate of the model. The numbers at the top indicate the number of features retained at various regularization strengths (λ). (B) LASSO regression coefficient path. This plot shows the logarithm of the regularization parameter (log(λ)) on the *x*-axis, where increasing λ corresponds to stronger regularization. The *y*-axis displays the coefficient size for each feature. DEG = differentially expressed genes, LASSO = least absolute shrinkage and selection operator.

**Figure 6.**
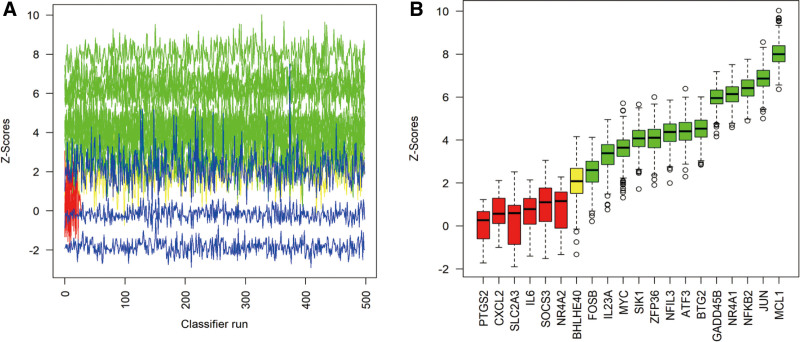
Gene selection using Boruta algorithm. (A) Feature distribution plot. (B) Boruta analysis result plot. The figure shows the *Z*-scores of each feature, which represent their importance.

To identify key genes associated with the OA phenotype, we performed an intersection analysis of DEGs, MEbrown module genes, and TNF signaling pathway-related genes. A Venn diagram (Fig. 4) showed that 20 candidate genes were shared among these gene sets. Then, we applied a LASSO regression model for variable selection. Ten-fold cross-validation was used to select the optimal λ, and the relationship between λ values and binomial deviance was plotted (Fig. 5A, B). We chose the model corresponding to λ_Ise to balance model simplicity and predictive performance, retaining 3 important genes. Boruta analysis categorized features into 3 groups: green (significantly useful), red (useless), and yellow (uncertain) (Fig. 6A, B). Thirteen significantly useful features (green) were selected. These features are crucial for predicting the target variable and provide a reliable basis for building predictive models.

Finally, by integrating the results from the LASSO regression and Boruta feature selection, we identified 4 core genes: MCL1, JUN, and GADD45B. These genes were consistently selected by both algorithms, highlighting their potential importance in the development and progression of knee osteoarthritis and their strong association with the TNF signaling pathway.

### 3.4. Results of core gene validation and immune infiltration analysis

**Figure 7.**
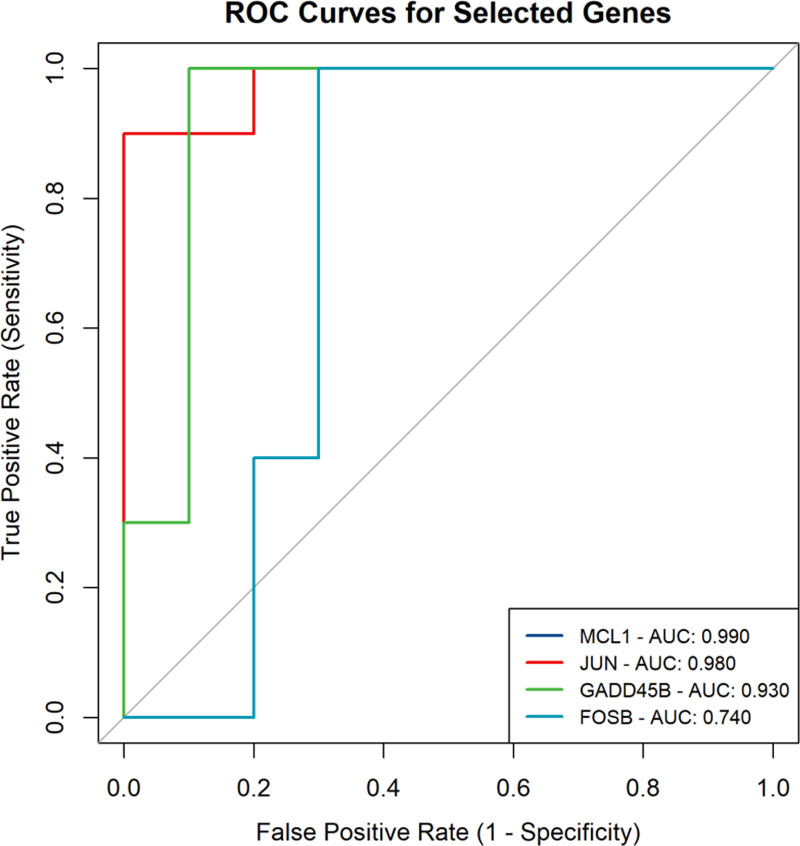
ROC validation of core gene. ROC = receiver operating characteristic.

We performed receiver operating characteristic curve analysis to evaluate the classification performance of the selected core genes (Fig. 7). The results indicated that MCL1, JUN, and GADD45B exhibited relatively high AUC values, highlighting their significant classification ability and potential as biomarkers for osteoarthritis. This further confirms their crucial roles in disease occurrence and progression.

**Figure 8.**
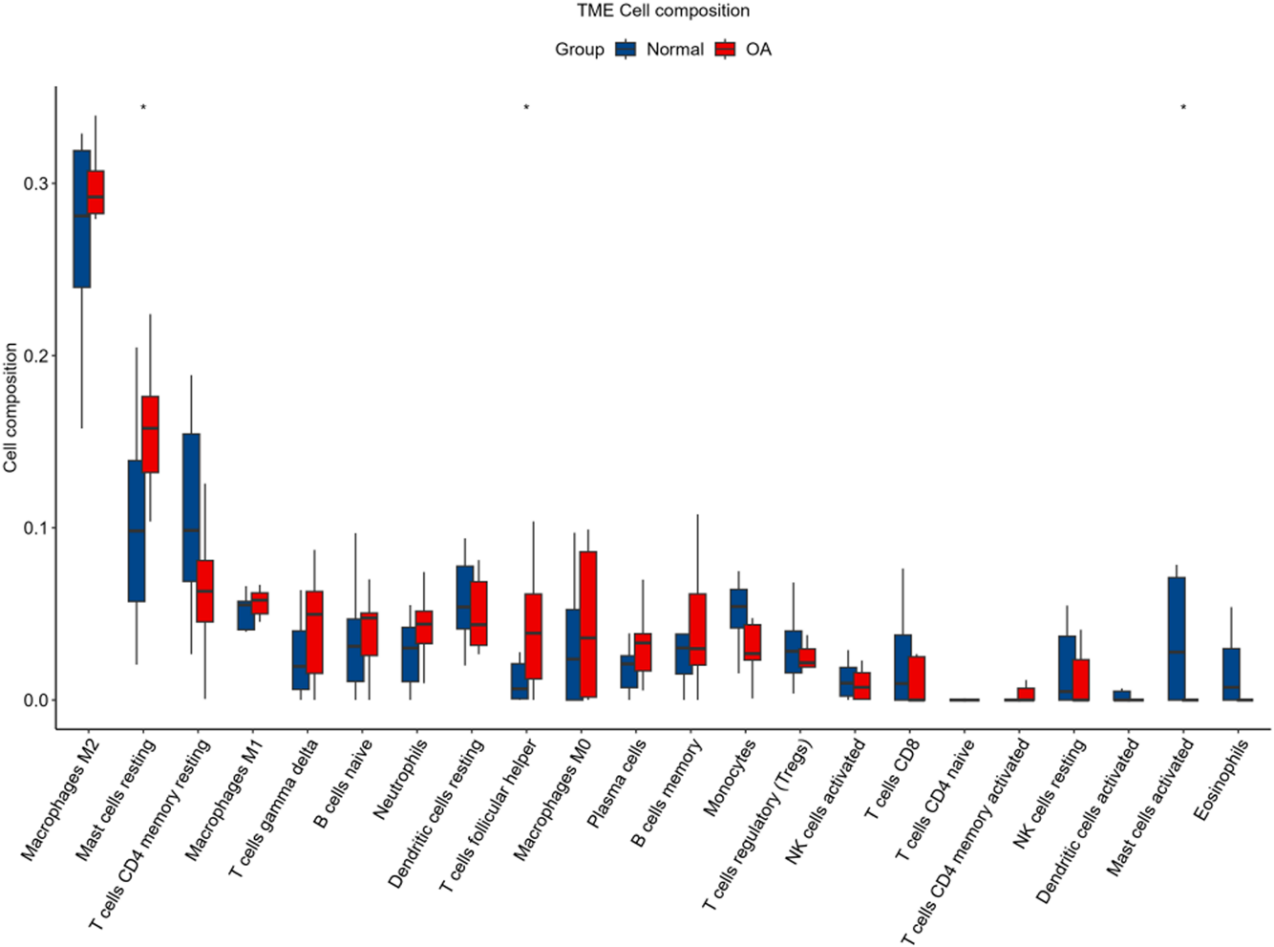
Immune infiltration box plot.

Immune infiltration analysis was conducted to assess immune cell distribution in the osteoarthritis microenvironment (Fig. 8). The results revealed notable differences in immune cell infiltration levels between osteoarthritis and normal samples, suggesting that specific immune cell infiltration may be associated with disease development. These findings provide a theoretical foundation for exploring potential immunotherapy targets.

## 4. Discussion

This study comprehensively explored the molecular mechanisms of osteoarthritis via diverse bioinformatics approaches, emphasizing the TNF signaling pathway and its related genes. The findings from differential expression, enrichment, network analyses and machine learning-based feature selection shed new light on osteoarthritis pathogenesis and offer a theoretical basis for personalized treatment strategies.

Differential expression analysis detected 222 DEGs, highlighting gene expression discrepancies between osteoarthritis and normal samples. These DEGs were predominantly enriched in inflammation-related pathways like the TNF signaling pathway and cytokine–cytokine receptor interaction pathway, with GO and KEGG analyses reinforcing their link to inflammatory responses and cytokine receptor binding. While prior studies have confirmed the TNF signaling pathway’s role in osteoarthritis, our GSEA results suggest its suppression in osteoarthritis samples is closely tied to disease-associated immune regulatory mechanisms. PPI network analysis also underscored the central role of TNF-related genes in signal transduction and inflammation regulation.

Moreover, WGCNA and intersection analysis pinpointed 20 potential key genes involved in the TNF signaling pathway. LASSO regression and the Boruta algorithm validated the significant predictive ability of genes such as MCL1, JUN, and GADD45B, with their high AUC values indicating potential as osteoarthritis biomarkers. Immune infiltration analysis revealed distribution differences of immune cells in osteoarthritis samples, further emphasizing the TNF signaling pathway’s importance in inflammation regulation and providing new avenues for immunotherapy research.

In OA synovial tissue, the JUN gene is abnormally expressed and linked to the p38-mitogen-activated protein kinase pathway. Persistent activation of this pathway causes synovial proliferation, inflammation and angiogenesis, driving OA progression.^[25]^ Analysis of multiple microarray datasets shows that the JUN gene is a key OA gene related to immune infiltration and is upregulated in OA synovial tissue. Further research indicates that MYC, JUN, DUSP1, and NFKBIA have specific expression in the immune system and potential as OA biomarkers and therapeutic targets.^[26]^ In OA chondrocytes, downregulated JUN expression induces chondrocyte senescence. Bioinformatics screening shows JUN is a key ASDEG in OA chondrocytes, with downregulated expression in OA rat models and senescent chondrocytes. SiRNA-Jun treatment suppresses chondrocyte proliferation and accelerates senescence, primarily by increasing chromatin instability.^[27]^ Moreover, studies show the JUN gene is involved in OA pathogenesis and can be a potential therapeutic target. Gallic acid phenyl ester, an active component in ZHUANGGUHUOXUETANG, has good binding activity with the JUN gene. It can reduce MMP3 and MMP13 expression in synovial fibroblasts induced by IL-1β and IL-6, showing promise in OA treatment.^[28]^

The role of the MCL1 gene is gaining increasing attention in the field of OA. Studies indicate that joint fixation procedures induce an increase in MCL1 gene expression in chondrocytes, suggesting that MCL1 may play a role in chondrocyte physiology or pathology.^[29]^ Additionally, research focusing on senescence-associated genes has identified key OA genes, with MCL1 emerging as a senescence-related marker, potentially linked to OA pathogenesis.^[30]^ GADD45B expression shows clear stage specificity in OA. GADD45B levels are higher in early-stage cartilage than in late-stage and normal donor cartilage, but lower in synovial tissue. Early OA cartilage has 2 times higher GADD45B expression than late-stage.^[31]^ Overexpression reduces COL2A1 promoter activity, enhances chondrocyte survival, and counteracts TNFα-induced death, with its transcription induced by NF-κB.^[31]^ However, it is important to note that research on the role of those genes in OA remains relatively limited. Further exploration is needed to clarify its mechanisms, functions, and clinical applications.

Although this study achieved important findings through integrative multi-method analyses, several limitations should be acknowledged. Initially, the analysis utilized publicly available datasets with a limited sample size. Expanding the sample size in future studies is crucial to strengthen result reliability. Secondly, the absence of functional validation experiments hinders a comprehensive understanding of the mechanisms underpinning the key genes. Additionally, bioinformatics analyses are contingent upon the completeness and accuracy of existing databases, which might lead to potential biases in certain results. Future research ought to incorporate experimental validation to delve deeper into the specific mechanisms of TNF signaling pathway-associated genes in osteoarthritis. It should also explore personalized treatment strategies based on these genes.

In conclusion, this study offers a systematic analysis of the role of TNF signaling pathway-related genes in osteoarthritis. It provides novel theoretical insights and scientific evidence that enhance our understanding of the disease mechanisms. Furthermore, it paves the way for potential personalized diagnostic and therapeutic strategies.

## Author contributions

**Conceptualization:** Shaoyang Zhai.

**Data curation:** Rui Wu.

**Formal analysis:** Shengzhen Fan.

**Investigation:** Ge Du.

**Methodology:** Xinkun Zhao.

**Project administration:** Weichen Huang.

**Resources:** Xinkun Zhao.

**Software:** Haoran Wan.

**Writing – original draft:** Shaoyang Zhai, Weichen Huang.

**Writing – review & editing:** Shaoyang Zhai, Weichen Huang.
